# PRC2‐Related Epigenetic Age Acceleration in Acute Myeloid Leukemia with *DNMT3A* and *IDH2* Mutations

**DOI:** 10.1002/adbi.202500710

**Published:** 2026-01-20

**Authors:** Zhengyi Yan, Luowei Yuan, Jinxing Wang, Shen Gu, Yong Lei

**Affiliations:** ^1^ School of Medicine The Chinese University of Hong Kong Shenzhen China; ^2^ Department of Pathology Technique Guangdong Medical University Shenzhen China; ^3^ School of Biomedical Sciences Faculty of Medicine The Chinese University of Hong Kong (CUHK) Hong Kong SAR China; ^4^ Gerald Choa Neuroscience Institute CUHK Hong Kong SAR China; ^5^ The Ciechanover Institute of Precision and Regenerative Medicine The Chinese University of Hong Kong Shenzhen China

**Keywords:** acute myeloid leukemia, DNA methylation, epigenetic aging, polycomb repressive complex 2

## Abstract

Aging is closely linked to epigenetic remodeling, with DNA methylation (DNAm) emerging as a robust biomarker for estimating epigenetic age (EA) and quantifying senescence. Dysregulation of aging‐associated DNAm has been implicated in diverse pathologies, including acute myeloid leukemia (AML). However, the effect of these epigenetic alterations in diseases and the underlying mechanism remains largely uncharacterized. Using causality‐enriched epigenetic clocks, we identified that adaptive DNAm dynamics are sensitive to short‐term therapeutic intervention in treating AML and may exhibit adaptive effects linked to better health outcomes. Subsequently, integrative genomic analysis showed significant associations between epigenetic aging and recurrent AML driver mutated genes, particularly *DNMT3A* and *IDH2*. The elevated adaptive aging associates with improved overall survival in cytogenetically normal AML harboring these mutations, highlighting its prognostic value in specific genomic contexts. Mechanistic analysis demonstrated that differentially methylated CpG sites in mutated gene‐specific AML subtypes are enriched at polycomb repressive complex 2 (PRC2) targets. These findings link mutation‐specific epigenetic aging, PRC2‐mediated methylation dynamics, and AML pathogenesis, offering insights into how aging‐related epigenetic dysregulation fosters malignant transformation. This study shows that AdaptAge can help reveal AML‑related DNAm dynamics when combined with genetic stratification, suggesting a path toward future biomarker development.

## Graphical abstract

This study reveals causality‑enriched epigenetic clocks offer additional insight into acute myeloid leukemia (AML) specific DNA methylation remodeling, particularly when integrated with genetic stratification, like *DNMT3A* or *IDH2* mutations. AdaptAge showed subgroup‑specific associations with survival and appeared responsive to treatment‑related changes, links this association with the altered methylation at polycomb repressive complex 2 (PRC2) targets.

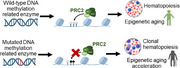

**Abbreviations**
AML: acute myeloid leukemia
CGI: CpG island
CH: clonal hematopoiesis
DMC: differentially methylated CpG
DNAm: DNA methylation
EA: epigenetic age
EAA: epigenetic age acceleration
ENCODE: the encyclopedia of DNA elements
OS: overall survival
PRC: the polycomb repressive complex
PRC2m: the DNA methylation level at the PRC2 targets
TCGA: The Cancer Genome Atlas

## 1 Introduction

Aging is a multifactorial process marked by progressive functional decline, driven in part by epigenetic remodeling across tissues. Among epigenetic modifications, DNA methylation (DNAm) has emerged as a robust biomarker of aging, enabling the estimation of the biological age of various tissues throughout the human lifespan [1, 2, 3]. The epigenetic clock (epi‐clock) represents a mathematical model that aggregates weighted DNAm levels from multiple CpG sites into what is known as epigenetic age (EA) [4, 5, 6]. The measure of epigenetic aging provided by epi‐clocks not only serves as an estimate of biological age but also quantifies the downstream consequences of aging mechanisms at the individual level [7]. EA acceleration (EAA) occurs when the EA deviates upward from chronological age, suggesting an accelerated aging process that may predispose individuals to various aging‐related conditions. This phenomenon has been linked to a range of phenotypes and pathologies, including a process characterized as clonal hematopoiesis (CH), where aged hematopoietic stem cells give rise to a population of blood cells with shared mutations, and various forms of cancer [8, 9, 10]. Such associations underscore the potential of DNAm as a biomarker for identifying individuals at greater risk for aging‐associated diseases and for developing targeted interventions aimed at precisely managing treatment.

Acute myeloid leukemia (AML) is a severe hematologic malignancy characterized by impaired differentiation of cells within the myeloid lineage [11]. Unlike many other cancers, de novo AML exhibits relatively fewer genetic mutations, typically with an average of 13 gene mutations per patient compared to the hundreds found in breast, lung, or pancreatic cancers [12]. Notably, approximately 45 % of these patients exhibit a cytogenetically normal (CN) profile [13], suggesting that typical oncogenic genetic traits, such as chromosomal rearrangements and genomic copy number variations, have a minimal impact on the evaluation of EAs in these AML subgroups [14]. Thus, CN‐AML provides a unique model for studying aberrant DNAm without the confounding effects of extensive cytogenetic alterations.

Among the most frequently mutated genes in AML are those involved in DNAm, such as *DNMT3A*, *IDH1/2*, and *TET2* [15]. These mutations usually disrupt the epigenetic landscape and cellular functions, contributing to AML pathogenesis. Early mutations like *DNMT3A* and *TET2* initiate clonal expansion, which can increase the risk of developing AML via CH [16]. Individuals with CH have a higher likelihood of progressing to AML due to the accumulation of mutations, like *IDH1* or *IDH2*, that can lead to malignant transformation [16, 17]. The presence of specific genetic mutations, such as *DNMT3A* R882 and *IDH2* R140, is commonly observed in various cohorts of AML, highlighting their potential significance in the disease. Generally, R882 mutants are associated with higher rates of treatment resistance and relapse, leading to statistically significant reductions in overall survival (OS) and event‐free survival compared to patients with *DNMT3A* non‐R882 mutations [15, 18]. To the contrary, *IDH2* mutation predominantly hotspot R140 correlates with a more positive clinical prognosis than other mutation sites, although their precise role in AML remains somewhat controversial [19, 20]. Intriguingly, gene mutations in AML have been linked with EA variations measured by a certain epi‐clock [21].

Most currently available epi‐clocks, like Horvath's Pan‐tissue clock, are based on the correlation between chronological age and CpG methylation [6]. Recently, new causality‐enriched epigenetic clocks have been developed, suggesting some age‐associated CpG sites may play a causal role in aging phenotypes [22], while others may only be byproducts of the aging process, challenging the common notion that most age‐related differential methylation sites are detrimental. These causality‐enriched epi‐clocks focus on CpG sites putatively causal for aging‐related traits, exemplified by models like AdaptAge and DamAge. These models distinguish between two opposing biological forces in aging: AdaptAge captures methylation changes at CpGs linked to adaptive mechanisms, which may confer resilience against or neutral age‐related decline, while DamAge reflects methylation alterations at CpGs associated with degenerative processes that drive functional deterioration and disease [22]. Subsequently, CausAge is defined as the epigenetic clock that combines both adaptive and damaging CpG sites. These causality‐enriched clocks hold the potential to provide deeper insights into DNAm alterations, enhancing our comprehension of EA in conditions like AML.

How aging‐related DNAm is involved in AML is largely unknown. Recent studies have revealed that aging‐associated DNAm regions are enriched at polycomb repressive complex 2 (PRC2) binding sites across various cell types [23]. PRC2 plays a pivotal role in histone modification and gene silencing during cellular differentiation and development [24]. In CH, a hallmark of both aging and AML, mutations in *DNMT3A* have been demonstrated to trigger altered methylation value at PRC2 target sites (PRC2m) [25]. These findings suggest that dysregulation of PRC2 caused by its aberrant binding landscape could serve as a potential causality bridge linking mutations in DNAm involved genes to altered EA in AML. Herein, we present evidence supporting the involvement of PRC2 in mediating AdaptAge in AML, particularly in the presence of specific epigenetic modifier mutations.

## 2 Results

### 2.1 Distinct EAs Predicted by Causality‐Enriched Epigenetic Clocks in AML

**FIGURE 1:**
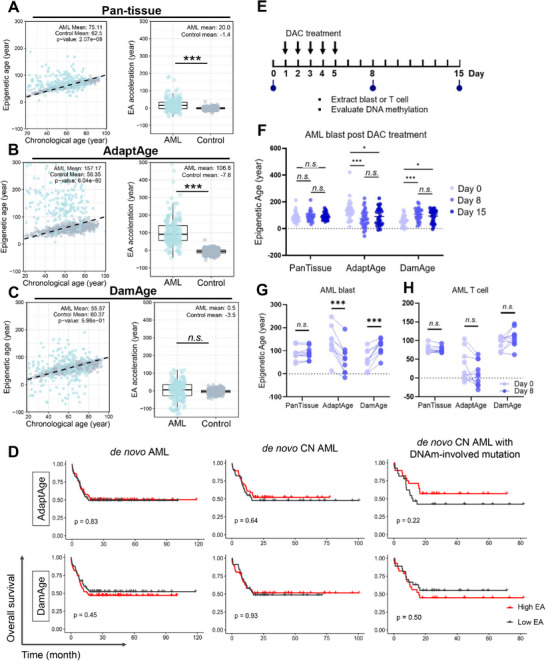
Alterations in epigenetic age metrics and clinical correlations of aging‐associated DNA methylation in AML. (A–C) Epigenetic age (EA) and epigenetic age acceleration (EAA) were estimated using a correlation‐based pan‐tissue clock and causality‐enriched clocks (AdaptAge and DamAge). Individual samples are plotted as dots (AML cohort: TCGA‐LAML, *n* = 194, blue; healthy controls: GSE40279, *n* = 633, gray). The dashed diagonal line (y = x) represents the theoretical equivalence between chronological and epigenetic age. (D) Kaplan–Meier survival curves for de novo AML patients stratified by median AdaptAge and DamAge values. (E) Schematic of serial isolation of myeloid blasts and autologous T cells from AML patients undergoing hypomethylating agent (HMA) therapy (GSE175758 cohort). (F) Longitudinal EA and EAA trajectories in AML blasts during HMA treatment (*n* = 28 samples). (G–H) Aging‐associated DNA methylation (DNAm) changes in AML blasts vs. paired autologous T cells of HMA therapy (*n* = 10 patient‐matched pairs). Intergroup comparisons were performed using two‐sided Mann‐Whitney U tests. Significance thresholds are denoted as follows: adjusted *p* ≤ 0.05 (^*^), adjusted *p* ≤ 0.001 (^**^), and "ns" (non‐significant; nominal *p* >0.05).

Previous studies showed that, although DNAm levels were increased in the AML as compared to normal blood, AML‐associated DNAm patterns, unexpectedly, do not correlate with patients' chronological age, underscoring the unique epigenetic landscape of the disease [21, 26]. To examine the implications of DNAm alterations in AML patients, we analyzed the EAs of 194 patients from The Cancer Genome Atlas (TCGA)‐LAML dataset in comparison to a control group (comprising 633 individuals aged between 19 and 101, see Methods), using broadly‐applied Horvath's Pan‐tissue (353‐CpGs) and the latest causality‐enriched clocks, respectively [5, 6, 22]. The assessment using the Pan‐tissue clock and CausAge revealed significant EAAs in AML patients compared to those of the control cohort [5], with mean EAAs of 20.0 and 29.5 years, respectively (Figure 1A; Figure S1; both adj. *p* < 0.001). These findings indicate that DNAm alterations in the AML cohort resemble those trends observed in older individuals. Previous study has successfully manifested the notion that DNAm changes can be either detrimental to cell functions or adaptive responses to molecular dysfunctions in the elderly [27]. To uncouple the deleterious and adaptive DNAm changes in AML patients, we used AdaptAge and DamAge to profile these alterations (Figure 1B,C). AML patients exhibited significantly elevated AdaptAge values compared with controls, with a mean EAA of 106.8 years (adj. *p* < 0.001). In contrast, the DamAge of AML patients did not exhibit a significant elevation or decline from that of the control group (*p* = 0.60). This finding suggested that substantial adaptive methylation shifts may indicate resilience to aging‐related decline in the AML patient with a high AdaptAge value. The broad spectrum observed in both AML AdaptAge and DamAge highlights a noteworthy divergence in the extent of aging‐related DNAm changes across the AML cohort.

To investigate the clinical relevance and prognostic sensitivity of adaptive methylation patterns, we stratified patients in the TCGA AML cohort into High AdaptAge and Low AdaptAge groups based on median values. We evaluated correlations between AdaptAge subgroups and probability of OS in three distinct populations: the full TCGA AML cohort, CN‐AML subgroups, and CN‐AML subgroups harboring common mutations in seven DNA methylation‐related genes (*DNMT1*, *DNMT3A*, *DNMT3B*, *IDH1*, *IDH2*, *TET1*, and *TET2*) [28]. In the DNAm‐related gene‐mutated CN‐AML subgroup, patients in the High AdaptAge group tended to exhibit higher OS compared to the Low AdaptAge group, compared to the other groups of full TCGA AML and CN‐AML, though this difference did not reach statistical significance (*p* = 0.22, log‐rank test). Parallel analyses were performed for DamAge, with patients stratified into High and Low DamAge groups using median thresholds. In contrast, the Low DamAge group also did not show improved OS relative to the High DamAge group with a statistically significant difference (*p* = 0.50, log‐rank test). While these findings suggest potential biological distinctions between AdaptAge and DamAge subgroups, neither metric demonstrated sufficient statistical power to independently predict favorable or unfavorable OS outcomes (Figure 1D).

### 2.2 AdaptAge and DamAge Are Sensitive to Capture the Short‐Term Medical Intervention

Notably, OS in AML is influenced by multifactorial determinants, including secondary cytogenetic abnormalities, treatment response, patient performance status, comorbidities, therapeutic regimens, and socioeconomic factors. To evaluate AdaptAge and DamAge as dynamic biomarkers in short‐term clinical interventions, we analyzed a distinct AML cohort treated with decitabine (DAC), a DNA‐hypomethylating agent (HMA) widely used in epigenetic therapy [29]. Peripheral blood samples (*n* = 28) were collected from patients undergoing a standardized 5‐day DAC regimen, and AML blasts were isolated for longitudinal DNA methylation profiling using Illumina 450K arrays at three time points: baseline (Day 0), Day 8, and Day 15 (Figure 1E). Both AdaptAge and DamAge demonstrated sensitivity to temporal DNAm changes during treatment. AdaptAge decreased significantly by Day 8 compared to baseline (Day 0; *p* < 0.001) and partially rebounded by Day 15, though it remained significantly lower than baseline levels (*p* < 0.05). Conversely, DamAge exhibited an inverse trend, increasing transiently at Day 8 before declining below baseline by Day 15. In contrast, the Horvath Pan‐tissue epigenetic clock failed to detect significant changes at either Day 8 or Day 15 (Figure 1F). These results suggest that the conventional epi‐clock, which relies on static methylation correlations, may lack sensitivity to short‐term therapeutic perturbations. AdaptAge and DamAge clocks dynamically reflect the acute effects of hypomethylating therapy.

To assess whether these dynamics were lineage‐specific or artifacts of global hypomethylation, we analyzed paired samples from AML blasts and T‐cells (lymphoid lineage) in a subset of patients (*n* = 10). Notably, AdaptAge and DamAge changes were restricted to myeloid lineage blasts, with no significant variations observed in T‐cells (Figure 1G,H). This lineage specificity underscores the potential utility of AdaptAge and DamAge in myeloid malignancies and excludes confounding effects by DAC‐induced global hypomethylation across blood cell types.

### 2.3 Adaptive Methylation Correlated With Evaluated Overall Survival in CN‐AML With *DNMT3A* and *IDH2* Mutations

**FIGURE 2:**
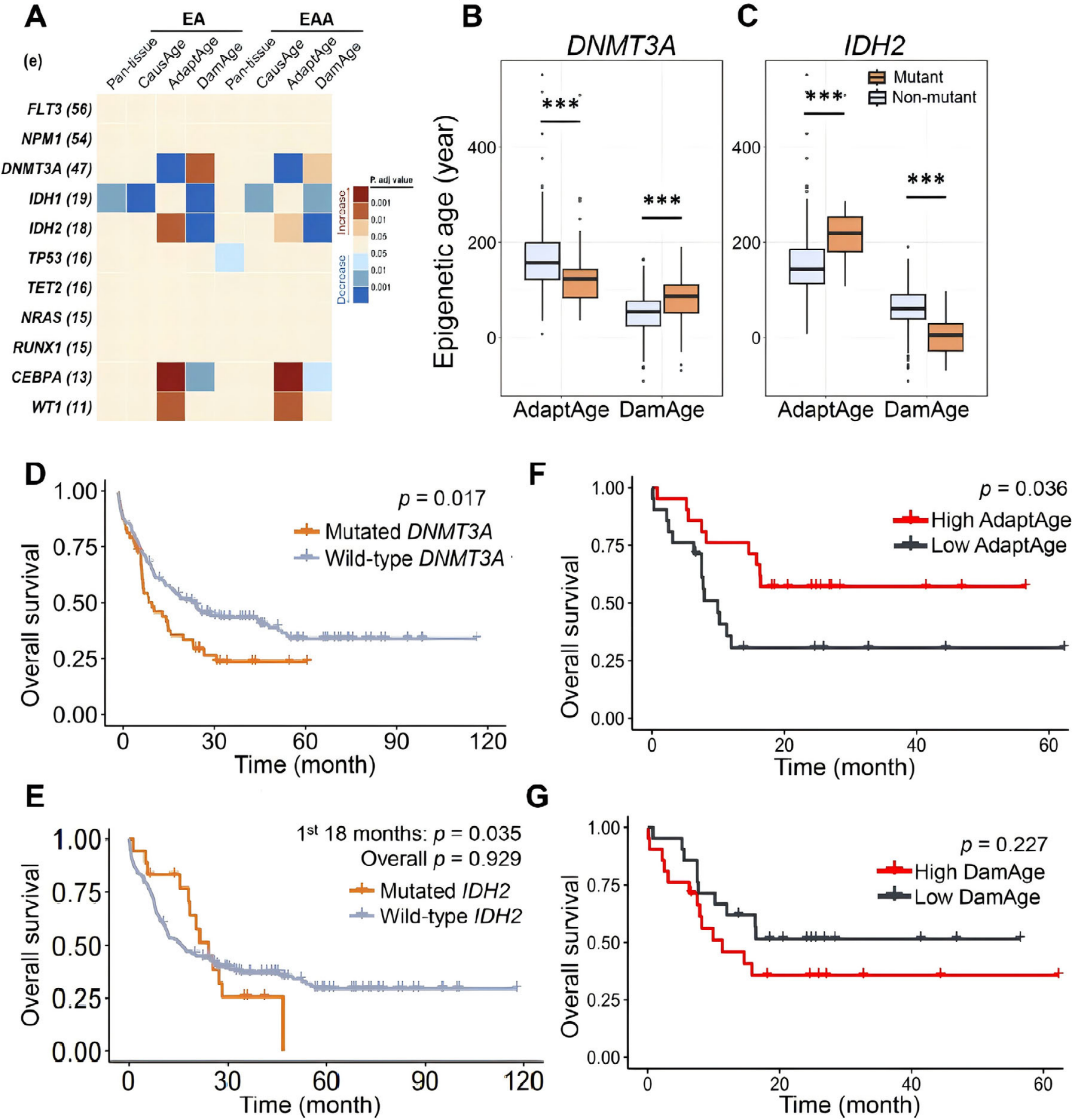
Genetics‐related epigenetic aging and survival outcomes in AML. (A) EA and EAA across AML patients stratified by the 11 most recurrently mutated genes (mutation frequency >10 cases; sample sizes per gene in parentheses). Group comparisons (mutant vs. wild‐type) were performed using two‐sided Mann‐Whitney U tests. (B,C) Differential AdaptAge and DamAge between *DNMT3A*‐mutant and *IDH2*‐mutant vs. wild‐type AML patients. (D,E) Kaplan–Meier survival curves comparing *DNMT3A*‐mutant (*n* =47) and *IDH2*‐mutant (*n* =18) AML patients to wild‐type cohorts. (F–G) Survival outcomes in cytogenetically normal AML (CN‐AML) patients stratified by epigenetic aging metrics: (F) *DNMT3A*‐mutant cases group, and (G) *IDH2*‐mutant cases grouped by high vs. low AdaptAge (median cutoff).

To explore the association between AdaptAge and DamAge values in AML patients with genetic mutations, we screened all statistically analyzable pathogenic variants (carrier number >2) in CN‐AML patients for differential analysis using stratification (Figure S2 and Tables S1 and S2). We observed distinct levels in AML patients carrying mutations in *DNMT3A*, *IDH2*, and *CEBPA* compared to non‐carriers (all adj. *p* < 0.05, Figure 2A). Other leading mutated genes recurrently identified in various AML cohorts [11, 30, 31], such as *FLT3*, *NPM1*, and *TP53*, exhibited no significant EA when assessed using the Pan‐tissue clock or CausAge predictions. This observation aligns with the notion that AML‐associated hypermethylation may not directly drive mechanisms underlying EA acceleration. Furthermore, other recurrently mutated genes implicated in DNA methylation regulation, such as *IDH1* and *TET2*, similarly showed no significant EA or EAA across the AdaptAge clocks evaluated in this study. These results suggest that while aberrant methylation is a hallmark of AML, not all methylation‐associated genetic alterations contribute to CpG methylation changes linked to aging‐related adaptive mechanisms in leukemogenesis. Since *DNMT3A* and *IDH2* encode proteins crucial for DNAm, and carrier numbers of their mutations were relatively large for statistical analysis (47 and 18, respectively), our subsequent analysis focused on patients harboring these genetic mutations.

It is revealed that AML patients with *DNMT3A* mutation exhibited significantly lower AdaptAge and elevated DamAge compared to non‐carriers, both indicating adverse DNAm changes (Figure 2B), indicative of a disrupted balance between protective epigenetic mechanisms and accumulating damage. To assess the biological impact of *DNMT3A* mutations on the longitudinal EA in AML, we compared survival outcomes between mutation carriers and non‐carriers. This adverse epigenetic profile aligned with their reduced OS (*p* = 0.02, log‐rank test, Figure 2D). *DNMT3A* mutations in AML are primarily concentrated at the R882 hotspot (Figure S3A), leading to a missense mutation that promotes leukemogenesis [32]. Our subsequent analysis showed that patients with *DNMT3A* R882 mutations demonstrated even more pronounced reductions in AdaptAge and increases in DamAge compared to those with non‐R882 *DNMT3A* mutations (*p* < 0.01, Figure S3B), suggesting that R882 variants exacerbate aging‐related epigenetic dysregulation.

In contrast, patients with *IDH2* mutations exhibited significantly higher AdaptAge and lower DamAge values compared to the non‐carriers, indicating more favorable DNAm changes (Figure 2C). The OS of patients with *IDH2* mutations was higher than that of those without *IDH2* mutation in the first 18 months of treatment after diagnosis (*p* = 0.04, log‐rank test, Figure 2E), suggesting a transient survival advantage during early treatment phases. These results are consistent with clinical statistics indicating that *IDH2*‐mutated AML patients often exhibit better initial responses to standard intensive chemotherapy [11, 33].

We next stratified de novo CN‐AML patients harboring *DNMT3A* or *IDH2* mutations into High and Low AdaptAge groups based on median values. Survival analysis revealed that patients with High AdaptAge exhibited significantly improved OS compared to those with Low AdaptAge (*p* = 0.04, log‐rank test) (Figure 2F). Although DamAge failed to distinguish survival outcomes when similarly stratified (*p* = 0.23), the Low DamAge patients exhibit a trend toward an improved OS relative to the High DamAge compared to that in the other groups of TCGA (Figure 1D,G). These findings demonstrate that elevated AdaptAge values correlate with favorable clinical trajectories in *DNMT3A*‐ and *IDH2*‐mutated AML. Overall, the divergent aging‐related epigenetic trajectories observed in *DNMT3A*‐ and *IDH2*‐mutated AML align with clinical OS and treatment outcome, suggesting the involvement of aging‐related DNAm in the mutation‐specific AML pathobiology.

To further investigate the relationship between the AdaptAge clock and clinical OS in AML patients stratified by frequent genetic mutations, we analyzed OS for recurrent AML‐associated mutations with sufficient cohort representation (sample size >10 per subgroup). Common mutations, including *FLT3*, *NPM1*, *IDH1*, *TET2*, and *NRAS*, showed no significant differences in OS between mutation carriers and non‐carriers (Figure S4). Similarly, these mutations had no discernible impact on AdaptAge values (Figure 2A). In contrast, *TP53* mutations—a well‐known tumor suppressor gene—were associated with markedly worse OS compared to *TP53* wild‐type patients (*p* < 0.001, log‐rank test), consistent with its role in driving aggressive disease progression. Notably, neither AdaptAge nor DamAge differed significantly between *TP53*‐mutated and wild‐type AML cases. This suggests that differential methylation patterns in *TP53*‐mutated AML lack enrichment at CpG sites causally linked to protective or damaging epigenetic aging effects [34]. These findings align with the observation that *TP53* mutations are relatively rare in de novo AML (5 %–10 % of cases) but enriched in therapy‐related AML (∼30 %), implying that *TP53* inactivation is not a prerequisite for AML initiation in most contexts [35]. Collectively, the AdaptAge exhibits a promoted sensitivity to the clinical OS, implying its potential utility in managing treatment and intervention for AML.

### 2.4 Cytogenetics‐Driven Epigenetic Complexity Substantially Compromised EA Prediction in AML

Chromosomal aberrations from AML exist [28]. The analysis of cytogenetic abnormalities is indispensable for AML, which represents the most important prognostic factor for patients with AML. To investigate whether recurrent cytogenetic aberrations correlate with aging‐related DNAm changes, we stratified the AML cohort into ten cytogenetic subgroups based on cytogenetic and molecular criteria in European LeukemiaNet recommendations [36] (e.g., CBFB::MYH11 fusion and MLLT3::KMT2A fusion) (Figure S5A). Using four epigenetic clocks (AdaptAge, DamAge, CausAge, Pan‐tissue), we compared EAs across these subgroups. No significant differences in EA were observed (ANOVA, adj. *p* >0.05 for all clocks), suggesting two potential explanations: (1) the existence of intra‐subgroup epigenetic heterogeneity, which makes it challenging to display any statistical differences within the current sample size. (2) The DNAm‐based epi‐clocks may lack resolution to capture epigenetic dysregulation rooted in structural chromosomal alterations (e.g., translocations, chromatin rearrangements). To further assess EA's clinical relevance, we classified the cohort into standard prognostic risk groups (favorable, intermediate, and poor). Subsequent analysis again revealed no significant AdaptAge or DamAge differences between risk categories (ANOVA, *p* >0.05), karyotype normality, or patient sex (Figure S5B–D). These results suggest that EA, as predicted by current DNAm‐based clocks, does not align with genetic risk stratification in AML. Applying epi‐clocks in AML with aberrant cytogenetics may need additional stratifications.

### 2.5 Alternated PRC2m Aligns With Adaptive Epigenetic Aging in AML

**FIGURE 3:**
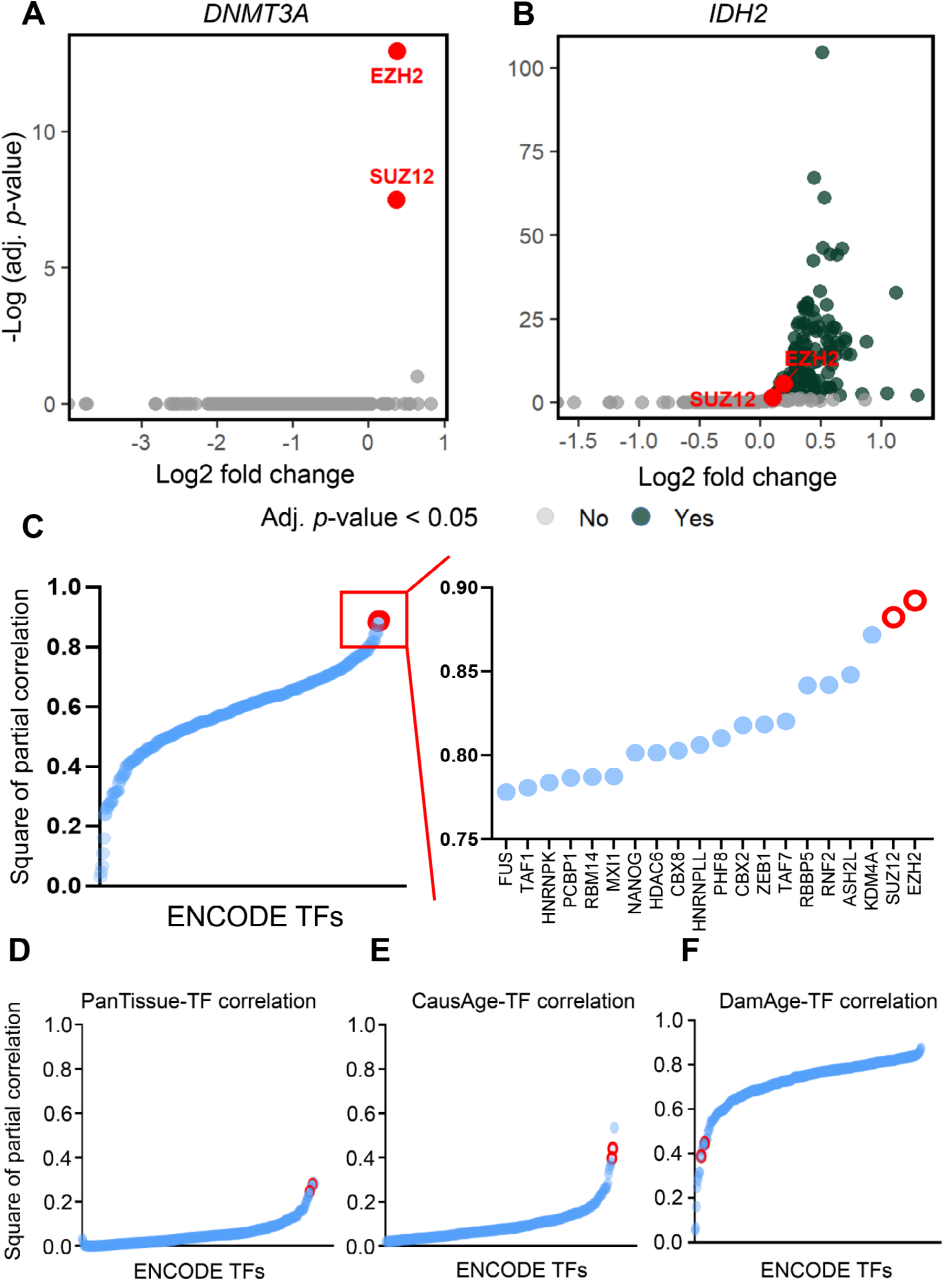
PRC2‐associated epigenetic aging signatures in AML. (A,B) Enrichment of transcription factor (TF) binding regions among differentially methylated CpGs (DMCs) in AML patients with (A) *DNMT3A* or (B) *IDH2* mutations (TCGA‐LAML cohort). Enrichment significance was evaluated via hypergeometric testing with false discovery rate (FDR) correction (FDR< 0.05). (C–F) Partial Pearson correlation analysis (adjusted for chronological age) between DNAm levels at ENCODE‐annotated TF binding sites and four epigenetic aging metrics. The top 20 TFs are ranked by absolute correlation coefficient. Red circles stand for EZH2 and SUZ12.

Given that DNAm plays a critical role in gene regulation by modulating the DNA binding of transcription factors (TFs), we aimed to identify TFs enriched in regulatory regions exhibiting DNAm‐associated perturbations. To achieve this, we performed enrichment analysis of 332 ENCODE‐derived TFs at differentially methylated CpGs (DMCs) in AML patients with *DNMT3A* or *IDH2* mutations, analyzing each mutation subgroup separately. Strikingly, only two TFs—EZH2 and SUZ12, core subunits of the Polycomb Repressive Complex 2 (PRC2)—showed significant enrichment (adj. *p* < 0.05) at DMCs in *DNMT3A*‐mutated AML (Figure 3A). A parallel analysis in the *IDH2*‐mutated cohort similarly identified EZH2 and SUZ12 among the most enriched TFs (Figure 3B). These findings suggest the involvement of PRC2 in the EA prediction in AML pathogenesis.

To validate these findings, we calculated Pearson correlation coefficients between the EAs predicted by four epigenetic clocks (AdaptAge, DamAge, CausAge, and Horvath's Pan‐tissue clock) and the DNAm values at ENCODE‐derived TF binding sites genome‐wide (Table S3). Notably, EZH2 (*Pearson r* ^2^ = 0.89) and SUZ12 (*Pearson r* ^2^ = 0.88) exhibited the highest partial correlation coefficients in relation to AdaptAge when controlling for the chronological age (Figure 3C). Other top correlated TFs include histone modifiers (KDM4A, ASH2L, RBBP5, PHF2, HDAC6), PRC1 subunits (RNF2, CBX2), and the genome integrity regulator ZEB1. EZH2 and SUZ12 are also listed among the top candidates with the highest correlation to Pan‐tissue and CausAge clocks. However, none of the correlation values exceed 0.50 (Figure 3D–F; Figure S6). The stronger association with AdaptAge highlights its unique linkage to PRC2‐mediated methylation dynamics in AML.

**FIGURE 4:**
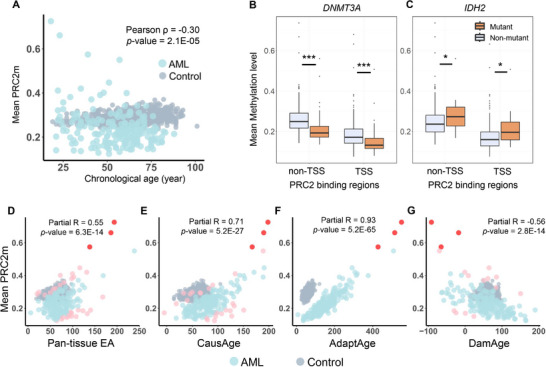
DNA methylation changes of PRC2 binding regions in AML. (A) The scatter plot for the relationship between chronological age and mean PRC2m in AML patients. (B,C) Mean values of CpG methylation within different PRC2 binding regions in AML patients with or without *DNMT3A* or *IDH2* mutation. TSS and non‐TSS regions are the PRC2 binding regions that contain a transcription start site (TSS) or do not. (D–G) Partial correlations (Pearson) between different epigenetic ages and mean PRC2m in AML patients whilst controlling for the chronological age. Each dot represents a patient. The pink color represents the correlation outliers of Pan‐tissue clock, CausAge, and DamAge, respectively; the red color represents the common correlation outliers of all three clocks (Mann–Whitney U test, ^*^ stands for *p*‐value ≤ 0.05, ^***^ stands for *p*‐value ≤ 0.001).

Methylation changes at PRC2 binding sites are associated with alterations in gene expression that can influence cellular aging processes [37, 38]. If aging‐related PRC2m patterns are observed consistently in AML patients, these changes could be associated with the patients’ chronological ages. However, we did not find a correlation (partial *r* = −0.30) between PRC2m and chronological ages (Figure 4A), indicating that PRC2m alterations in AML are driven by leukemogenic processes rather than normal aging. To further investigate PRC2m's role, we compared PRC2m levels in AML patients with *DNMT3A* or *IDH2* mutations. Patients with *DNMT3A* mutations exhibited significantly reduced PRC2m (*p* < 0.001; Figure 4B), whereas those with *IDH2* mutations showed elevated PRC2m (*p* < 0.05; Figure 4C), echoing the roles of DNAm‐associated gene mutations in aging‐related epigenetic dysregulation (Figure 2B,C).

Although no significant correlation between PRC2m and chronological age in AML patients was detected (Figure 4A), PRC2m was negatively correlated with DamAge (partial *r*< −0.5) and positively correlated with EA value of Pan‐tissue clock, CasuAge, and AdaptAge (partial *r* >0.5, Figure 4D–G), whilst controlling the effects of chronological age. The strongest association was observed with AdaptAge (partial *r* = 0.93), implying that PRC2 plays an important role in the adaptive response of the epigenetic aging in AML pathogenesis. To rule out confounding by AML‐associated global hypermethylation at PRC2 targets, we compared CpG sites in PRC2 targets with those in the four epigenetic clocks. Only 0.2 % of CpG sites overlapped between PRC2 targets and AdaptAge (4/2472) or DamAge (4/2562) clocks (Figure S7). Moreover, outliers across these epi‐clocks showed minimal overlap (only 3/49 shared across all four clocks; Figure 4D–G). These results indicate that AdaptAge could capture specific adaptive effects via the PRC2 pathway rather than nonspecific methylation changes.

### 2.6 Gain of Adaptive Epigenetic Aging at PRC2 Targets in AML

**FIGURE 5:**
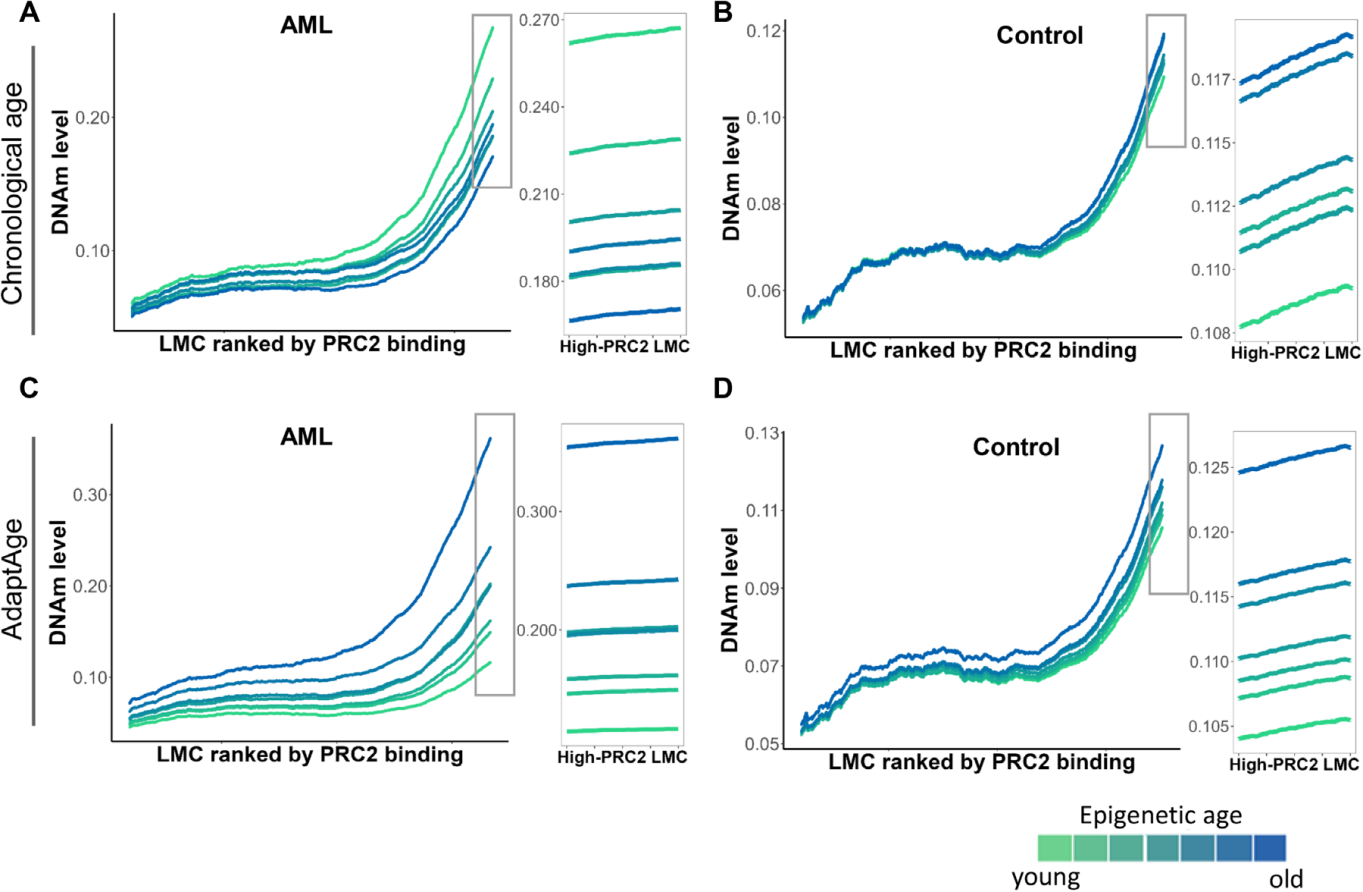
DNA methylation dynamics at low‐methylated CpG regions (LMCs) stratified by PRC2 occupancy. (A,B) Mean DNA methylation at PRC2‐bound LMCs in the control cohort (GSE40279, *n* = 633), stratified into seven age‐matched groups (equal sample size) based on chronological age deciles or AdaptAge levels. (C,D) Analogous analysis in the de novo AML cohort (TCGA‐LAML, *n* = 194), stratified by chronological age or AdaptAge. PRC2‐bound LMCs were defined by overlapping EZH2 and SUZ12 ChIP‐seq peaks (ENCODE data).

Previous studies have demonstrated that low‐methylated regions bound by PRC2 in embryonic stem cells exhibit age‐associated DNAm gains in human peripheral blood mononuclear cells [22]. To investigate whether this phenomenon is conserved in AML, we analyzed DNAm patterns by ranking PRC2‐bound low‐methylated regions and calculating their mean methylation levels across AML samples. Strikingly, in contrast to non‐AML controls, the AML cohort did not display the expected progressive gain of DNAm at PRC2 targets using chronological age nor pan‐tissue epi‐clock (Figure 5A,B; Figure S8). Thus, the consistent gain of DNAm at PRC2 targets with age observed in aging studies was not evident in the AML cohort. Intriguingly, certain younger AML patient groups showed higher methylation gains at these regions compared to older subgroups—a trend inconsistent with observations in healthy populations and prior studies [22]. To resolve this discrepancy, we substituted chronological age with AdaptAge. A clear and consistent age‐associated methylation gain at PRC2 targets emerged in both AML and control cohorts (Figure 5C,D). Furthermore, AdaptAge improved the resolution of epigenetic aging stratification, enabling distinct differentiation between age subgroups within the AML cohort. This enhanced sensitivity extended even to weakly PRC2‐bound loci in AML, which previously lacked discernible age‐related methylation patterns. These findings suggest that AdaptAge outperforms pan‐tissue epi‐clock in evaluating EAA in AML.

## 3 Discussion and Conclusions

Aging is a nonlinear and heterogeneous process characterized by the progressive deterioration of physiological function. Previous advances in epigenetics have enabled the quantification of biological age through DNAm patterns at specific CpG sites. Notable models include Horvath's pan‐tissue clocks [6, 39], Hannum's blood‐based clock [5], PhenoAge [40], and various epi‐clocks [41, 42] that correlate methylation states with age‐related outcomes across diverse diseases, including genetic disease [43, 44], neuropathologies [45], and certain cancers [46]. However, these tools are based primarily on associations and are trained on healthy populations, which limits their applicability in disease contexts. A paradigm shift has emerged with the development of causality‐enriched epigenetic clocks, such as AdaptAge and DamAge, which distinguish adaptive from deleterious methylation changes during aging [22]. This study offers a potential framework to examine how epigenetic dysregulation relates to disease phenotypes.

AML, particularly CN‐AML, provides a unique model for studying epigenetic clocks due to its relatively low mutational burden and the high prevalence of epigenetic‐related gene mutations. CN‐AML constitutes approximately 50 % of de novo AML cases [13], characterized by the absence of chromosomal abnormalities yet frequent mutations in genes such as *DNMT3A* and *IDH1/2*. The predominance of such mutations positions AML as an informative setting to evaluate whether causality‐enriched clocks capture disease‑relevant methylation dynamics. This interest aligns with recent therapeutic studies showing that anti‑aging drugs such as metformin may modulate leukemogenesis in DNMT3A‑mutant contexts [47, 48]. Considering that CH‑associated mutations exert diverse and sometimes opposing effects on hematopoietic stem cell behavior [49, 50], a refined measure of epigenetic aging may eventually help interpret differential therapeutic responses.

Because chronological age is only an approximation of physiological aging, we examined the relevance of AdaptAge in genetically stratified AML subgroups. Higher AdaptAge values emerged in CN‐AML patients with *DNMT3A* and *IDH2* mutations subgroup, suggesting improved OS. Interestingly, *DNMT3A* mutation usually causes global hypomethylation in AML [16], while the *IDH* mutations result in a hypermethylation phenotype in AML [51]. These observations suggest that AdaptAge may capture aspects of mutation‑linked epigenetic remodeling beyond global methylation shifts.

Beyond prognostication, we evaluated the clocks’ sensitivity to therapeutic intervention. Longitudinal analysis of peripheral blood blasts from AML patients treated with DAC revealed that AdaptAge and DamAge dynamically tracked therapy‐induced epigenetic remodeling, whereas traditional clocks and matched T‑cell controls showed little to no response. These patterns suggest that causality‑enriched clocks may be sensitive to disease‑specific DNAm remodeling. Additional datasets will be required to determine whether such responsiveness reflects meaningful biological or clinical processes.

Mechanistically, AdaptAge's prognostic power may relate to its association with PRC2m. The mispositioning of PRC has been reported in a number of hematological malignancies, including AML [52]. In *DNMT3A*‐mutant AML, reduced PRC2m levels, potentially secondary to global hypomethylation, disrupt PRC2 positioning at CpG island (CGI), impairing gene silencing and hematopoietic differentiation. A recent study demonstrated that *DNMT3A* mutations lead to CGI hypermethylation at PRC targets, supporting the notion that altered PRC2m are from a combination of global and focal methylation changes in AML [53]. Conversely, *IDH2*‐mutant AML exhibits elevated PRC2m and confers a less aggressive phenotype. These divergent PRC2m profiles align with clinical outcomes [33]. Although these patterns parallel the subgroup‑specific AdaptAge survival associations, our findings remain correlative and do not establish a mechanistic link. The PRC2 targets identified were derived from ChIP‐seq ENCODE data originating from cell types distinct from primary hematopoietic stem and progenitor cells, which may introduce a partial localization shift of transcription factor binding profiles. Experimental studies will be required to determine whether AdaptAge captures functional interactions between DNAm and PRC2 activity.

This study provides a systematic exploration of epigenetic aging in AML through high‐resolution profiling of mutation‐associated DNAm patterns across a clinically annotated cohort. A key strength lies in its inclusion of statistically powered patient subgroups for recurrent mutations (e.g., *DNMT3A* and *IDH2*), enabling robust associations between EAA and molecular subtypes. However, several limitations warrant consideration. First, the TCGA‑LAML cohort (n = 194) provides only moderate power for subgroup analyses and lacks comprehensive therapy‑specific annotation. Second, although the clocks used here are designed to be tissue‐agnostic, their performance in malignancies with aberrant cell populations (e.g., AML blasts) warrants further investigation, ideally using single‑cell DNAm profiling. Third, the DNAm data obtained from Human Methylation 450K BeadChip (∼2 % of CpGs) presents methodological constraints, limiting the comprehensiveness of DNAm analyses. Fourth, the presence of inconsistent outlier individuals with extremely advanced chronological ages across various epigenetic clocks was observed in both our study and AML cell lines (e.g., KG1A and HL60) [6]. These outliers highlight that EA reflects a composite index of biological processes—including clonal selection and therapy‐induced stress—rather than a direct surrogate for chronological age. Larger cohorts with longitudinal samples will be essential to delineate how specific mutation types (e.g., *DNMT3A* R882 vs. non‐R882) and functional impacts (loss‐of‐function, dominant‐negative) modulate EA trajectories [18, 54].

Integrating expanded DNAm coverage with PRC2‑AgeIndex, chromatin accessibility, and other multi‑omics data may help clarify the biological significance of aging‑related methylation patterns in AML.

In summary, our findings provide exploratory evidence that causality‑enriched epigenetic clocks offer additional insight into AML‑specific DNAm remodeling, particularly when integrated with genetic stratification. While AdaptAge showed subgroup‑specific associations with survival and appeared responsive to treatment‑related changes, mechanistic details remain unresolved. These results highlight potential directions for future research into the role of disease‑linked aging signatures in AML, pending validation in larger cohorts.

## 4 Experimental Section

### 4.1 AML Samples and Control

DNA methylation data of the whole blood sample and clinical data of 194 de novo AML patients’ whole blood were downloaded from the‐The Cancer Genome Atlas (TCGA)‐LAML dataset using R (version 4.3.2). DNA methylation level was quantified as the beta value measured by HumanMethylation450K BeadChip. Genetic mutation and survival data for the TCGA‐LAML cohort were sourced from cBioPortal (Table S1).

DNA methylation data of the whole blood samples from 656 control individuals were obtained from the GSE40279 dataset in the Gene Expression Omnibus (GEO) using R (version 4.3.2) in RStudio (version 2023.12.0) [5]. Similarly, DNA methylation data of the whole blood sample and clinical data of 194 de novo AML patients’ whole blood were downloaded from the‐The Cancer Genome Atlas (TCGA)‐LAML dataset. DNA methylation level was quantified as the beta value measured by HumanMethylation450K BeadChip. Genetic mutation and survival data for the TCGA‐LAML cohort were sourced from cBioPortal (Table S1) [55]. Following data normalization, outlier detection and removal for the 656 control samples were performed using principal component analysis (PCA) in Python (version 3.10.4) [5]. Each sample was converted into a z‐score based on the squared distance from the population mean along the first principal component. The z‐scores were then adjusted to false discovery rates using the Gaussian cumulative distribution function and the Benjamini–Hochberg procedure [56]. Samples with a false discovery rate below 0.2 were identified as outliers and excluded. This filtering process was iteratively repeated until the sample values were within the −20–20 range, resulting in a final dataset of 633 samples.

### 4.2 Data Normalization

Each epigenetic clock employed normalization when calculating the epigenetic age for both control and AML samples. Specifically, the Pan‐tissue clock utilizes the BMIQ method for normalizing DNA methylation data [6, 57]. In contrast, the causality‐enriched epigenetic clocks do not apply normalization to the beta values of DNA methylation data during the calculation of epigenetic age [22] (https://bio‐learn.github.io/). In the study of PRC2m, AML samples were not compared or merged with the control group; therefore, the batch effect was assessed exclusively within the AML samples and the normal samples. The analysis revealed that there is no significant batch effect, indicating that there was no need to correct for batch effects within the AML samples (TCGA 2013). For the probes of the Illumina Infinium 450k DNA methylation assay, we focused exclusively on those necessary for epigenetic age calculations using epigenetic clocks and those located within PRC2 binding regions. None of those probes was removed from the analysis.

### 4.3 Epigenetic Age and Epigenetic Age Acceleration Calculation

To estimate the epigenetic age of our samples, we employed the Horvath clock, developed in 2013 (pan‐tissue clock). This clock uses a set of 353 CpG sites to calculate a weighted average of methylation levels, which is then transformed into epigenetic age using R (version 4.3.2). The raw methylation data from the 450 K array were preprocessed and processed based on the R scripts provided by Horvath's original publication [6]. In parallel, we applied the causality‐enriched epigenetic clocks (CausAge, AdaptAge, and DamAge) using the algorithm provided [22].

The formula used for calculating the epigenetic age acceleration (EAA) is as follows:

EAA=EpigeneticAge−ChronologicalAge

Be noted that, unlike the correlation observed between epigenetic age and chronological age in the general population, this relationship is not linear in the AML cohort. Therefore, using a residual‐based method, which relies on a linear correlation, may not be suitable for calculating epigenetic age acceleration in AML patients. We are not confident in generating a linear model based on the TCGA AML cohort, which could precisely represent the epigenetic age acceleration. Therefore, we choose the method of age acceleration difference in its reliability, herein.

### 4.4 Analysis to Identify PRC2 Genomic Binding Sites

ENCODE ChIP‐seq data were downloaded from UCSC databases [58], using the R script based on the Regulatory Element Interrogation Script [59]. Methylation levels within these regions were assessed by the beta values of CpG sites located within the reference genome GRCh37/hg19. The PRC2 binding regions were identified by overlapping the regions of the EZH2 and SUZ12 peaks. Regions containing at least one transcription start site (TSS) within the PRC2 binding area were designated as TSS regions. The promoter regions of genes were defined as ‘upstream = 3000, downstream = 200′ to overlap with the PRC2 binding regions.

The mean value of the DNA methylation at the PRC2 target regions (PRC2m) is calculated using the following formula, where CpG site refers to that locates within the PRC2 binding region.

$$mPRC2m=\frac{\sum CpG\;site\;methylation}{N_{CpG\;site}}$$

The selection of mean methylation at the PRC2 target is referred to following reference [23], which has been shown to improve age prediction accuracy and robustness.

### 4.5 Differential Analysis, Association Outlier Detection, and Visualization

Since the distribution of epigenetic ages and epigenetic age acceleration in the control samples is not normal, differential analysis was conducted using the Mann–Whitney U test (two‐sided) in R (version 4.3.2) in RStudio (version 2023.12.0). The association outlier identification method applied in this study involves a regression‐based approach. Specifically, a linear model was fitted where the independent variable is used to predict the dependent variable. An outlier threshold was established as twice the standard deviation. Data points with residuals exceeding this threshold were flagged as outliers. Survival curves were produced using cBioPortal.

### 4.6 Enrichment of ENCODE Transcription Factor Target Region in *DNMT3A* and *IDH2* Mutated De Novo AML Cohort's Differentially Methylated CpGs

DNA methylation beta values from Illumina HumanMethylation450 arrays were obtained from TCGA‐LAML. Mutation status (binary classification: mutated vs. wild‐type) for *DNMT3A* and *IDH2* was annotated for all samples. Transcription factor (TF) binding regions were derived from ENCODE ChIP‐seq datasets (Txn_Factr_ChIP_E3), encompassing 338 TFs aligned to the hg19 reference genome. Genomic annotations included CpG probe positions (FDb.InfiniumMethylation.hg19) and transcription start site (TSS) coordinates (UCSC hg19).

For differential methylation analysis, beta values were logit‐transformed to M‐values to stabilize variance, and methylation differences between *DNMT3A*‐ or *IDH2*‐mutated and wild‐type groups were assessed via linear modeling using the *limma* package. Statistically significant differentially methylated CpGs (DMCs) were defined by Benjamini–Hochberg false discovery rate (FDR)‐adjusted *p* < 0.05. TF enrichment analysis involved intersecting CpG probes with TF binding regions, followed by hypergeometric testing to evaluate DMC enrichment against a genome‐wide background (Table S4). TFs with fewer than five overlapping CpGs were excluded to mitigate spurious associations. Significant TFs were identified after FDR correction (*p* < 0.05).

## Author Contributions

Contribution: Y.L. and S.G. conceived the study, secured funding, and managed overall direction. Y.L. and Z.Y. designed experiments. Z.Y., L.Y., and J.W. analyzed data. Z.Y., S.G., and Y.L. took the lead in writing the manuscript. All authors contributed edits and provided critical feedback that helped shape the final manuscript.

## Conflicts of Interest

All authors declare no conflict of interest.

## Supporting information




**Supporting File 1**: adbi70085‐sup‐0001‐SuppMat.docx

**Supporting File 2**: adbi70085‐sup‐0002‐TableS1.xlsx

**Supporting File 3**: adbi70085‐sup‐0003‐TableS2.xlsx

**Supporting File 4**: adbi70085‐sup‐0004‐TableS3.xlsx

**Supporting File 5**: adbi70085‐sup‐0005‐TableS4.xlsx

## Data Availability

The data set of Hannum et al. is available in GEO (GSE40279). The data that support the findings of this study are openly available in the TCGA‐LAML dataset at (https://doi.org/10.1056/nejmoa1301689), in cBioPortal at (https://doi.org/10.1158/0008‐5472.can‐23‐0816), and in ENCODE at (https://doi.org/10.1038/nature11247) respectively.
