# Supplementary material for: PRC2‐Related Epigenetic Age Acceleration in Acute Myeloid Leukemia with DNMT3A and IDH2 Mutations
Source: Adv Biol (Weinh). 2026 Jan 20;10(1):e00710. doi: 10.1002/adbi.202500710 (PMC12817234; doi:10.1002/adbi.202500710)
Supplement: Supplementary file 1 — Supporting File 1: adbi70085‐sup‐0001‐SuppMat.docx [file ADBI-10-e00710-s003.docx]

Supporting Information for

PRC2-related Epigenetic Age Acceleration in Acute Myeloid Leukemia with *DNMT3A* and *IDH2* Mutations

Zhengyi Yan^1^, Luowei Yuan^1^, Jinxing Wang^2^, Shen Gu^3,4*^, Yong Lei^1,5*^

^1^ School of Medicine, The Chinese University of Hong Kong-Shenzhen, Shenzhen, China

^2^ Department of Pathology Technique, Guangdong Medical University, Shenzhen, China

^3^ School of Biomedical Sciences, Faculty of Medicine, The Chinese University of Hong Kong, Hong Kong SAR, China

^4^ Gerald Choa Neuroscience Institute, CUHK, Hong Kong SAR, China

^5^ The Ciechanover Institute of Precision and Regenerative Medicine, The Chinese University of Hong Kong-Shenzhen, Shenzhen, China

**Article type**: Original Research Article

**Correspondence:** [leiyong@cuhk.edu.cn](mailto:leiyong@cuhk.edu.cn) (Y.L.), shengu@cuhk.edu.hk (S.G.)


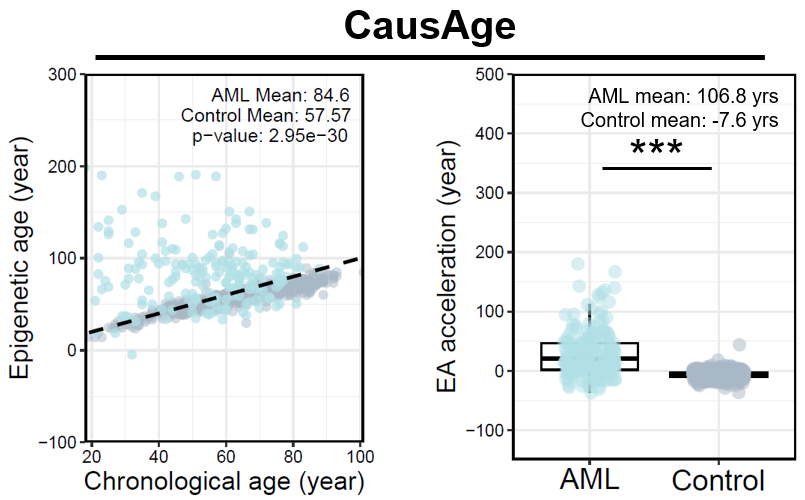


**Figure S1:** Epigenetic age (EA) and epigenetic age acceleration (EAA) estimated by causality-enriched clocks. Group difference was evaluated using two-sided Mann–Whitney U test; *** indicates adjusted p ≤ 0.001. Each dot represents an individual sample (AML cohort from TCGA-LAML, n = 194, blue; healthy people cohort as controls from GSE40279, n = 633, gray). The dashed line denotes the identity line (y = x).


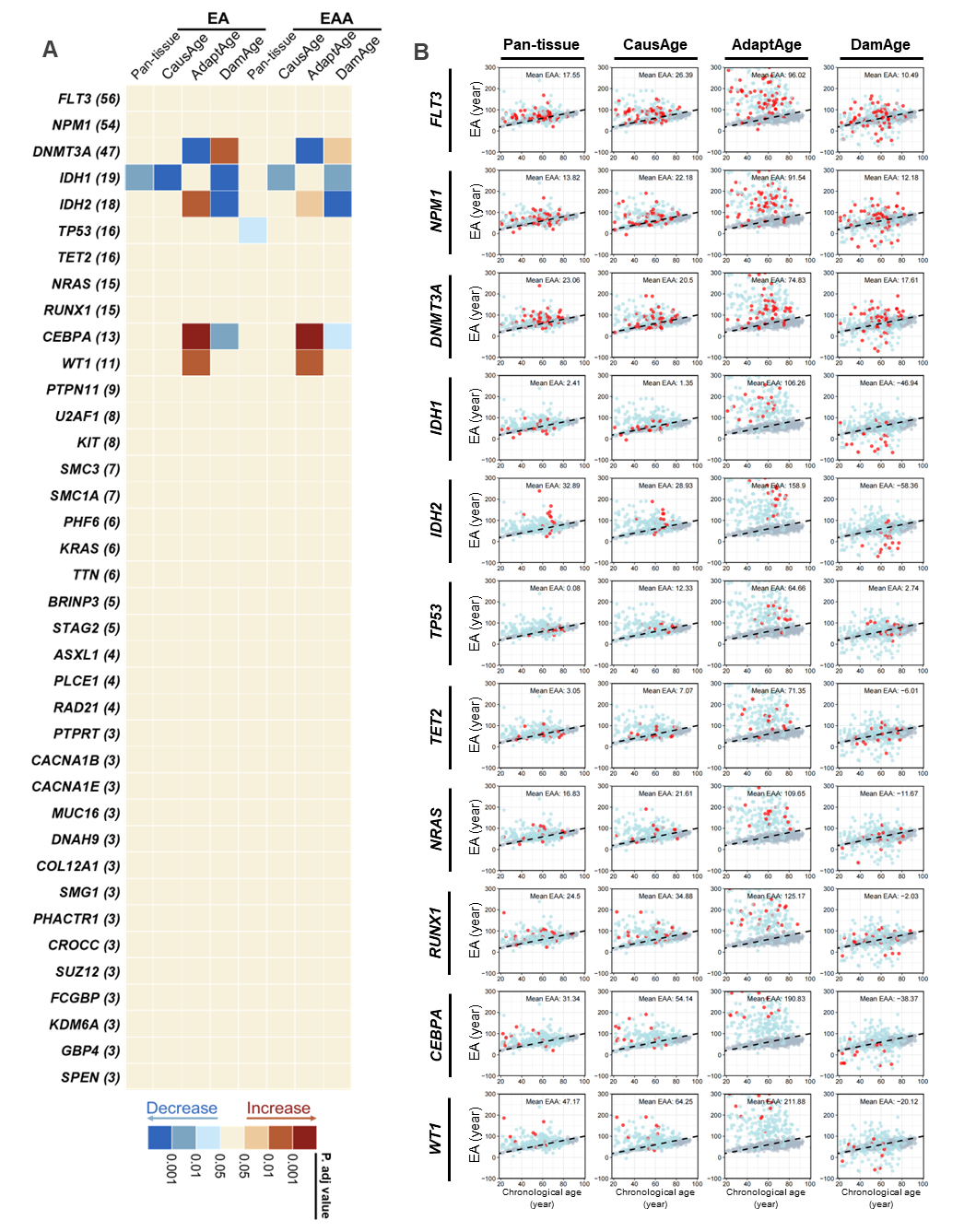


**Figure S2:** Difference analysis of epigenetic aging across mutation‐defined subgroups in the TCGA-LAML cohort. (A) Comparative analysis of EA and EAA in AML patients stratified by mutated genes (mutation frequency > 2; sample sizes shown in parentheses). Group-wise comparisons were performed by two-sided Mann–Whitney U tests. (B) Scatterplots of EA versus chronological age, stratified by status of 11 most recurrently mutated genes. Each dot represents an individual AML patient (AML cohort from TCGA-LAML, n = 194, powderblue; the patient with the certain genetic mutation is shown as red; healthy people cohort as controls from GSE40279, n = 633, gray).


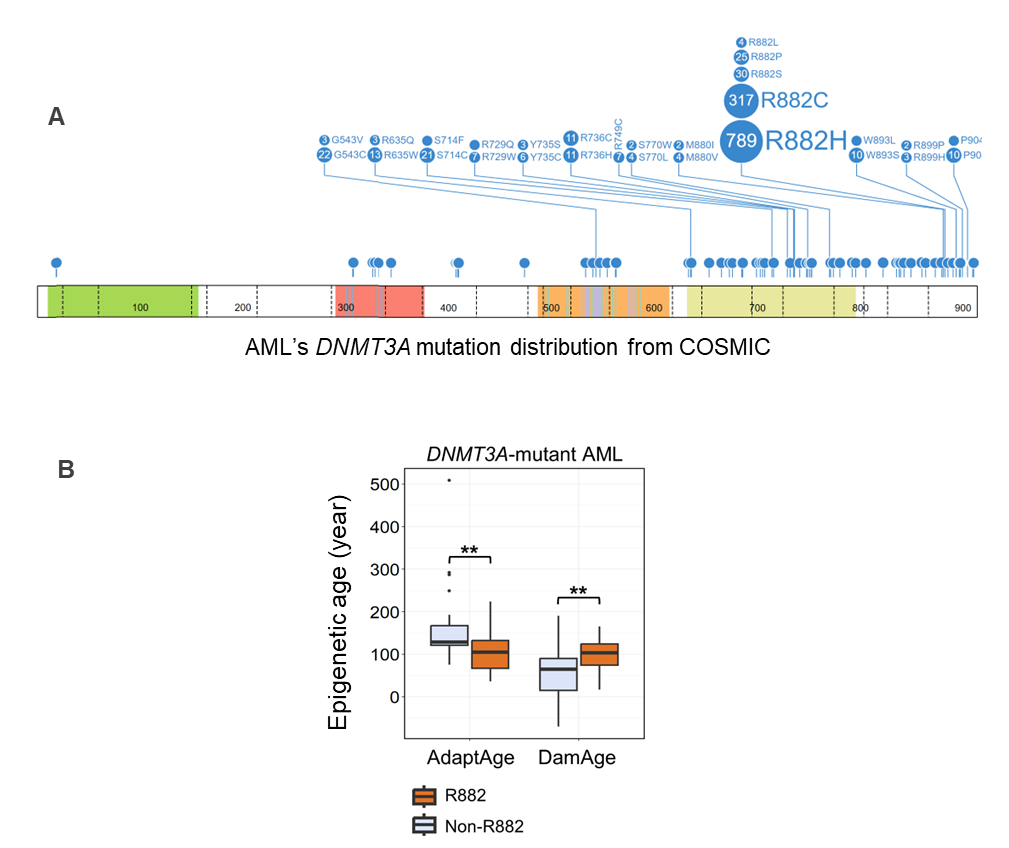


**Figure S3:** *DNMT3A* mutation spectrum and epigenetic aging differences by different mutation spots. (A) Distribution of *DNMT3A* mutations observed in the acute myeloid leukemia cohort from COSMIC, which was visualized by St. Jude Clould (https://pecan.stjude.cloud/variants/proteinpaint?gene=DNMT3A). Mutation numbers in hotspots were shown in blue circles. (B) Boxplots comparing AdaptAge and DamAge between AML patients harboring R882 *DNMT3A* mutations and those with non-R882 *DNMT3A* variants. Group differences were assessed using two-sided Mann–Whitney U tests with ** indicating adjusted p ≤ 0.01.


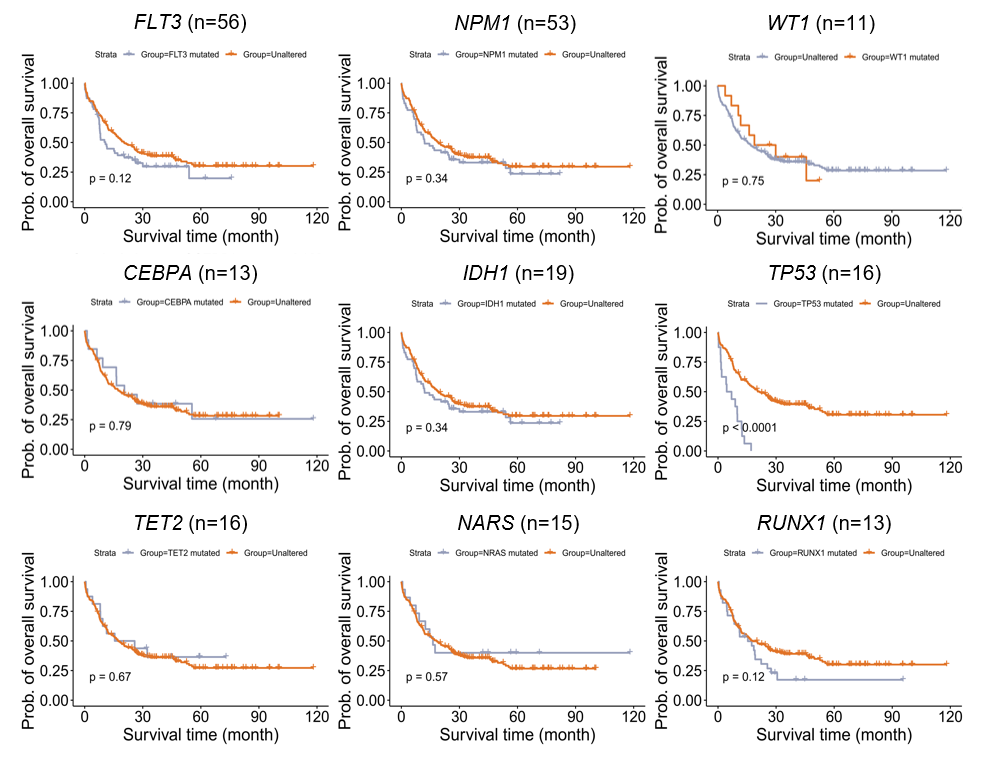


**Figure S4:** Kaplan–Meier analysis of overall survival in the de novo TCGA-LAML cohort, stratified by mutation status for each recurrently mutated gene with sufficient carrier frequency. Patients are grouped by the presence or absence of the indicated mutation, and survival curves are compared using the log-rank test (p-values shown).


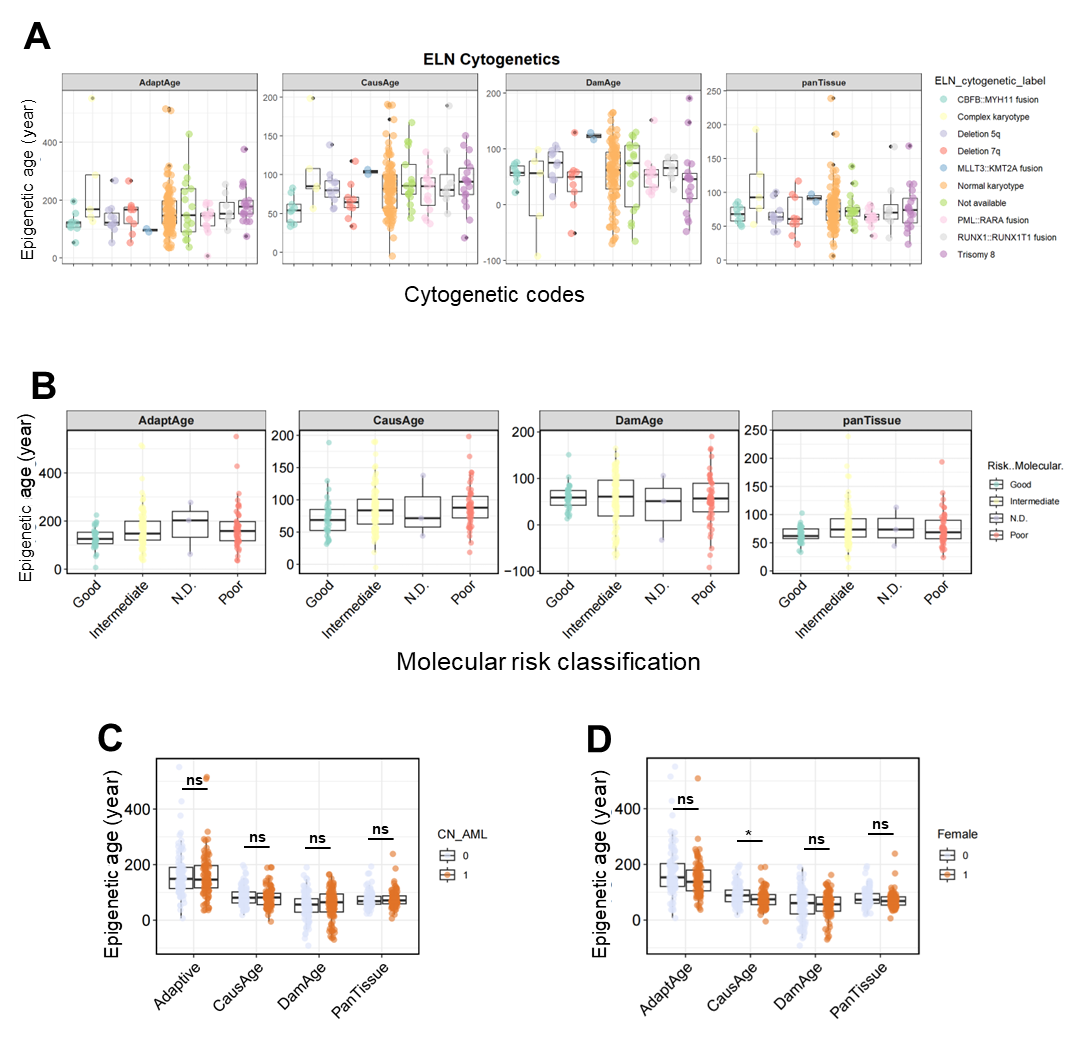


**Figure S5:** Association between clinical parameters and epigenetic age (EA) in the de novo TCGA-LAML cohort. *P*-value is adjusted using the Bonferroni correction method. One-way ANOVA was used to evaluate differences in EA across patient subgroups: (A) Cytogenetic subgroups categorized based on European LeukemiaNet recommendations. (B) Cytogenetic risk categories. (C) Karyotype normality (cytogenetically normal vs. abnormal AML). (D) Patient sex (male vs. female).


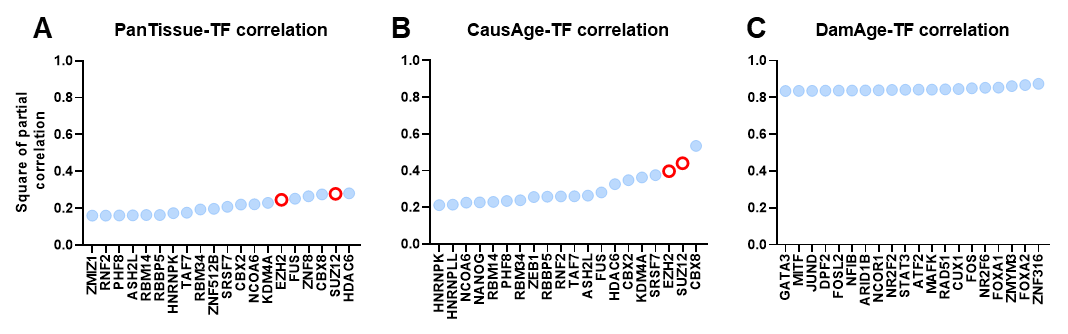


**Figure S6:** Top 20 ENCODE transcription‐related proteins exhibiting the strongest partial correlations with epigenetic age metrics in AML. Partial Pearson correlations were calculated between DNA methylation levels at TF binding sites and each epigenetic age measure, controlling for chronological age. (A) Horvaths Pan‐tissue epigenetic age. (B) CausAge (combined causal clock). (C) DamAge (damaging aging).


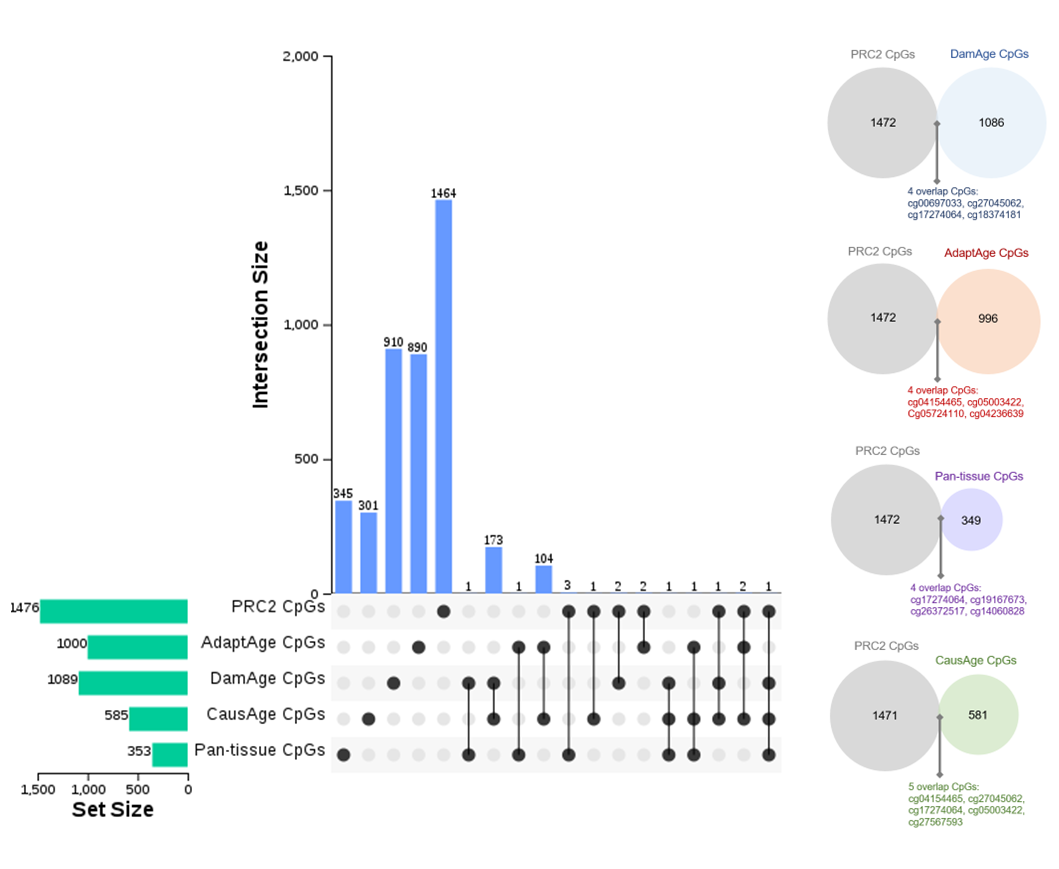


**Figure S7:** UpSet Venn diagrams depicting the overlap between CpG sites located within PRC2 target regions and those incorporated into various epigenetic clocks. PRC2 CpG: CpG sites mapped to PRC2 binding regions; Pan-tissue CpG: CpG sites employed in Horvath’s pan-tissue clock; DamAge CpG: CpG sites utilized by the damaging‐aging clock (DamAge); CausAge CpG: CpG sites included in the combined causal clock (CausAge); AdaptAge CpG: CpG sites used by the adaptive‐aging clock (AdaptAge).


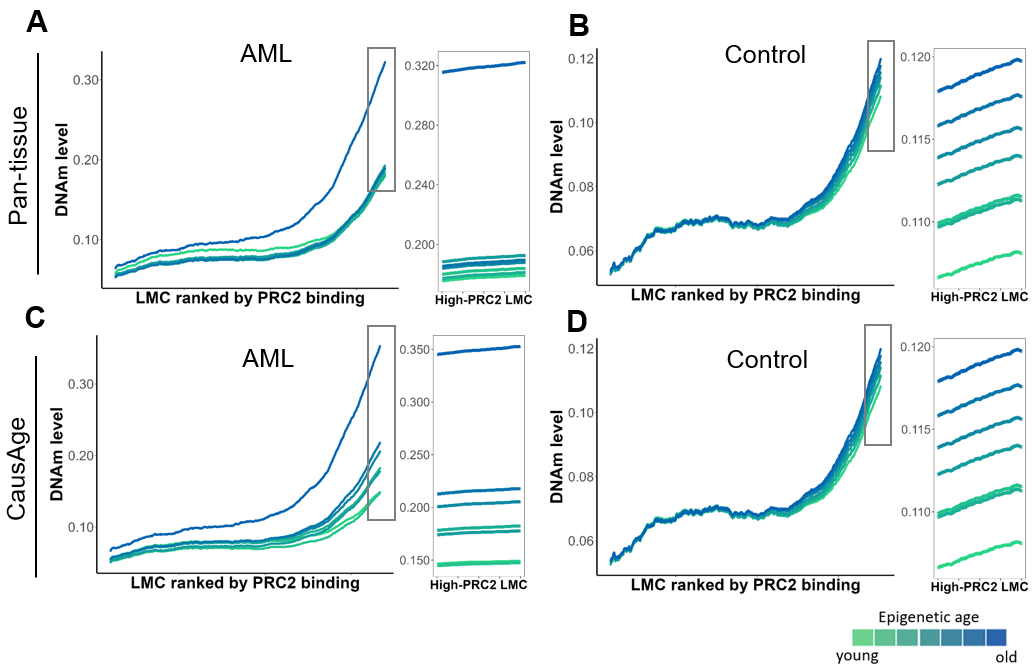


**Figure S8:** DNA methylation dynamics at low‐methylated CpG regions (LMCs) stratified by PRC2 occupancy. PRC2 binding intensity was defined by overlapping EZH2 and SUZ12 ChIP–Seq peaks from ENCODE H1 embryonic stem cells. 9A) Mean DNA methylation at PRC2‐LMCs across seven groups (equal sample size) of pan-tissue EA in the TCGA-LAML cohort. (B) Mean DNA methylation at PRC2‐LMCs across seven groups (equal sample size) of CausAge EA levels in the TCGA-LAML cohort. (C) Mean DNA methylation at PRC2‐LMCs across seven groups (equal sample size) of pan-tissue EA deciles in the healthy control cohort (GSE40279, n = 633). (D) Mean DNA methylation at PRC2‐LMCs across seven groups (equal sample size) of CausAge EA levels in the healthy control cohort.

**Table S1**: Genetic mutation and survival data for the TCGA-LAML cohort. Sourced were adapted from cBioPortal (<https://www.cbioportal.org/>).

**Table S2**: The mutated gene profile in the TCGA-LAML cohort. Whether the mutated gene is a cancer gene is determined using OncoKB (<https://www.oncokb.org/>).

**Table S3**: Partial Pearson correlations between four epigenetic ages and mean DNA methylation levels within protein binding regions in the TCGA-LAML cohort, controlling for chronological age. For each binding region, the table reports the correlation coefficient (r) and Benjamini–Hochberg–adjusted p-value for chronological age, Horvath's pan-tissue clock, AdaptAge, DamAge, and CausAge.

**Table S4**: Enrichment of DNA methylation changes at ENCODE‐defined transcription factor (TF) binding regions among differentially methylated CpGs (DMCs) in AML patients with *IDH2* mutations. Hypergeometric tests were performed to assess overrepresentation of DMCs within each TF binding region, and p-values were adjusted for multiple comparisons using the Benjamini–Hochberg method.
